# Ceramide synthase-6 confers resistance to chemotherapy by binding to CD95/Fas in T-cell acute lymphoblastic leukemia

**DOI:** 10.1038/s41419-018-0964-4

**Published:** 2018-09-11

**Authors:** Dattesh Verlekar, Sung-Jen Wei, Hwangeui Cho, Shengping Yang, Min H. Kang

**Affiliations:** 1 Cancer Center, School of Medicine, Texas Tech University Health Sciences Center, Lubbock, TX 79430 USA; 2 Department of Pediatrics, School of Medicine, Texas Tech University Health Sciences Center, Lubbock, TX 79430 USA; 3 Department of Cell Biology and Biochemistry, School of Medicine, Texas Tech University Health Sciences Center, Lubbock, TX 79430 USA; 4 Department of Pathology, School of Medicine, Texas Tech University Health Sciences Center, Lubbock, TX 79430 USA; 5 Department of Internal Medicine, School of Medicine, Texas Tech University Health Sciences Center, Lubbock, TX 79430 USA

## Abstract

Ceramide synthases (CERS) produce ceramides which are key intermediators in the biosynthesis of complex sphingolipids and play an important role in cell proliferation, differentiation, apoptosis and senescence. CERS6 is an isoform of ceramide synthases known to generate ceramides with C16 acyl chain (C_16_-Cer). CERS6 and C_16_-Cer levels were significantly higher in acute lymphoblastic leukemia (ALL) cells in comparison to peripheral blood mononuclear cells and T lymphocytes derived from healthy human volunteers. We investigated the role of CERS6 in chemo-resistance in T-ALL cell lines. Stable knockdown of *CERS6* in CCRF-CEM and MOLT-4 cells resulted in increased sensitivity to ABT-737, a pan-BCL-2 inhibitor, while CCRF-CEM cells with exogenous *CERS6* expression showed resistance to ABT-737 relative to the vector control. The cytotoxic activity of ABT-737 in *CERS6* knockdown cells was significantly reduced by the addition of a caspase-8 inhibitor Z-IETD, suggesting that CERS6 alters the cytotoxicity via extrinsic pathway of apoptosis. By co-immunoprecipitation of CERS6 in CCRF-CEM cells, we identified CD95/Fas, a mediator of extrinsic apoptotic pathway, as a novel CERS6 binding partner. In Fas pull-down samples, FADD (Fas-associated protein with death domain) was detected at higher levels in cells with *CERS6* knockdown compared with control cells when treated with ABT-737, and this was reversed by the overexpression of *CERS6*, demonstrating that CERS6 interferes with Fas–FADD DISC assembly. CERS6 may serve as a biomarker in determining the effectiveness of anticancer agents acting via the extrinsic pathway in T-ALL.

## Introduction

Acute lymphoblastic leukemia (ALL) is the most common childhood and adolescent cancer, and approximately 2900 new cases of pediatric ALL are diagnosed annually in the United States^1^. Sixty percent of the cases occur at less than 20 years^2^ and the survival rate of childhood ALL is close to 90%^3,4^. Although much progress has been made in understanding cellular responses to standard chemotherapeutic agents, approximately 10% of pediatric patients with ALL do not respond to treatment, and ultimately die of the disease^3,4^. As cancer cells continue to evolve mechanisms to circumvent stressors leading to development of intrinsic or acquired drug resistance, a significant percentage of patients with standard-risk and high-risk ALL relapse, and post-relapse treatment rarely results in long-term survival^5^. Mortality in high-risk disease is about 35%^6^ and the treatment of infants and adults still needs improvement^4^.

**Fig. 1:**
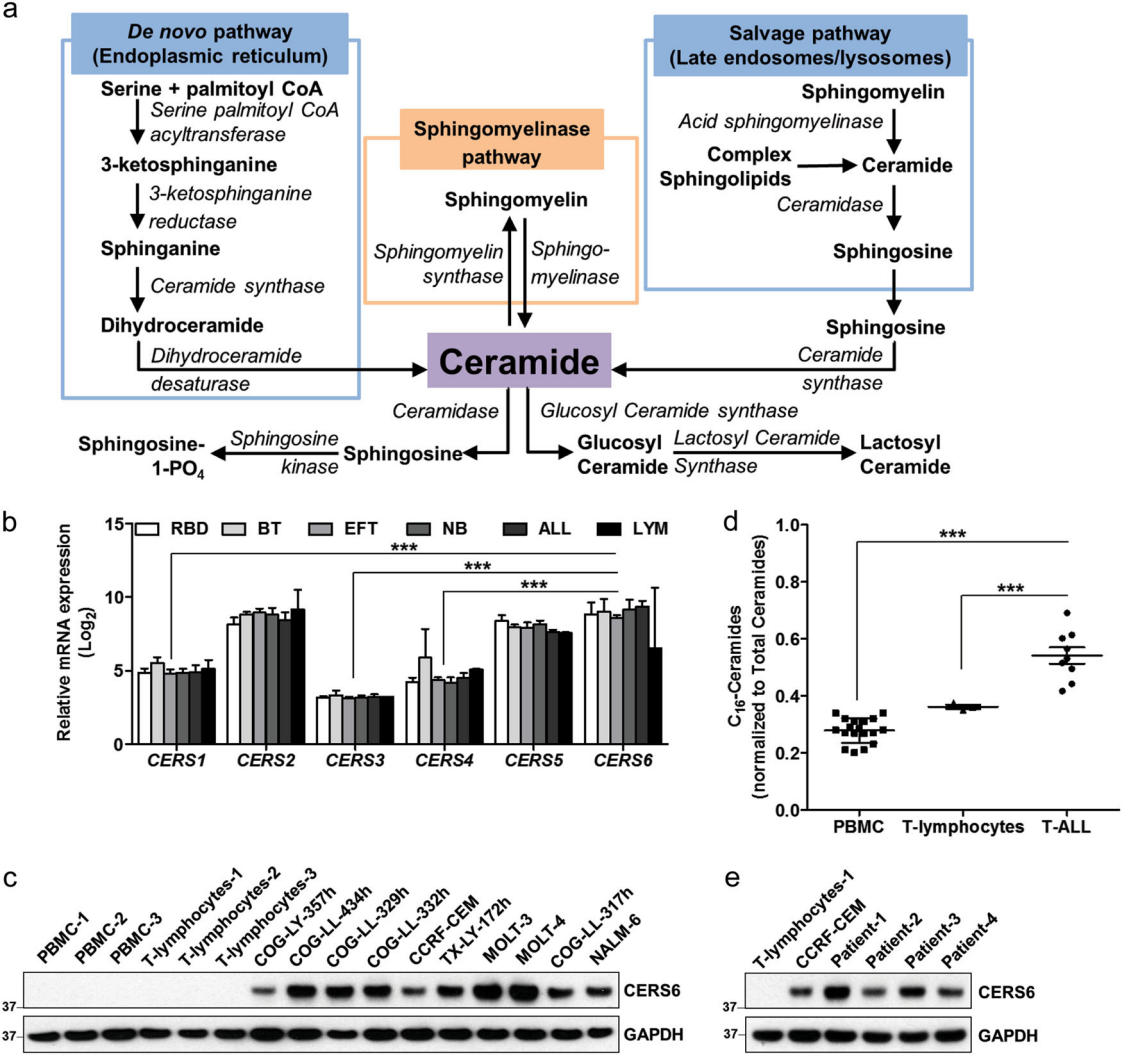
CERS6 is overexpressed in ALL. **a** Biosynthesis and metabolism of ceramides. Ceramides are generated either de novo or via the salvage pathway. They are metabolized to sphingosine or may serve as substrates for the synthesis of glucosyl ceramides, lactosyl ceramides or sphingomyelins. Sphingomyelins degrade to ceramides via the sphingomyelinase pathway and are synthesized from ceramides via sphingomyelin synthase. **b** mRNA expression of the six ceramide synthase isoforms in the NCI PPTP panel of 23 cell lines comprising six different pediatric cancers. RBD rhabdomyosarcoma, BT brain tumor, EFT Ewing’s family of tumors, NB neuroblastoma, ALL acute lymphoblastic leukemia, LYM lymphoma. The mRNA expression was determined within the NCI Pediatric Preclinical Testing Program. **c** CERS6 protein expression in acute lymphoblastic leukemia (ALL) cell lines in comparison to peripheral blood mononuclear cells (PBMCs) and T lymphocytes obtained from blood of healthy human volunteers. GAPDH was used as a loading control. **d** C_16_-Ceramides in T-cell ALL cells in comparison to PBMCs and T lymphocytes. Ceramide levels were quantitated by HPLC/MS/MS (PBMC: 0.28 ± 0.04, *n* = 18; T lymphocytes: 0.36 ± 0.01, *n* = 3; T-ALL: 0.54 ± 0.09, *n* = 9). **e** CERS6 protein expression in T lymphocytes isolated from primary lymphoid malignancy samples in comparison to normal T lymphocytes *** *p* < 0.001

Sphingolipids are bioactive lipids and primarily include sphingosines, ceramides and sphingomyelins that control a variety of cellular functions^7^. Many chemotherapeutic drugs are reported to modulate sphingolipid pathways, though their contribution to cytotoxicity is controversial. Ceramides, key intermediates in the biosynthesis of all the complex sphingolipids, have a significant role in the regulation of cell growth, differentiation, apoptosis and senescence^8^. Six mammalian ceramide synthase (CERS1–6) homologs use a relatively restricted subset of acyl-Coenzyme A for ceramide synthesis by N-acylation of the sphingoid base^9^. CERSs produce ceramides using either the de novo synthesized sphinganine^10^ or by acylation of sphingosines from the salvage pathway (Fig. 1a). Ceramides are also generated by breakdown of sphingomyelins using sphingomyelinases.

The expression levels of CERSs vary between different tissues. *CERS1* is predominantly expressed in the brain and skeletal muscles in mouse^11^. While *CERS2* is ubiquitously expressed, kidney, liver and intestine are the major tissues with high *CERS2* expression^11^. *CERS3* shows highest expression in testis and *CERS4* is not specific to any particular tissue. *CERS5* has been extensively studied and is the main CERS found in the lung epithelia^7^. *CERS6* is expressed in almost all tissues, but shows a very low expression profile in each of them, except for small intestine^11^. CERS6 predominantly synthesizes C16-ceramide (C_16_-Cer) and is subcellularly localized mainly in the endoplasmic reticulum^12^, and also in the plasma membrane^13^. *CERS6* displays homology with *CERS5*^14^ and *CERS6* knockout mouse shows a high reduction in C_16_-Cer levels in most tissues^15^. Knockdown of *CERS6* in colon adenocarcinoma cells caused a decrease in C_16_-Cer and protected the cells against tumor necrosis factor-related apoptosis-inducing ligand (TRAIL)^16^, whereas C_16_-Cer generated by *CERS6* in human head and neck squamous cell carcinoma (HNSCC) cells increased tumor development and growth^17^, suggesting an anti-apoptotic role of CERS6 in tumor cells.

Treatment of pediatric ALL includes remission induction therapy for 4–5 weeks, consolidation therapy for 4–8 weeks, delayed intensification therapy for 8–9 weeks sandwiched between two interim maintenance therapies for 8 weeks each, followed by maintenance therapy for 2–3 years, and is the longest phase of treatment^18,19^. Glucocorticoid is one of the backbone regimen for the treatment of pediatric ALL. ABT-737 is a small molecule inhibitor of B-cell lymphoma 2 (BCL-2) family of proteins^20^, which binds directly to the hydrophobic groove of anti-apoptotic BCL-2, B-cell lymphoma-extra large (BCL-X_L_) or BCL-w promoting the oligomerization of BAX and BAK to induce apoptosis^21^. Although ABT-737 as a single agent has shown substantial activity in multiple myeloma^22^, it can significantly enhance the activity of ALL standard-of-care treatment drugs like vincristine, L-asparaginase and dexamethasone in vitro and in vivo^23^. A recently developed modified BCl-2 inhibitor, ABT-199, shows high selectivity towards BCL-2 without inhibiting BCL-X_L_ or BCL-w and is currently approved for chronic lymphocytic leukemia.

The purpose of this study is to understand the roles of ceramides in cancer and to define sphingolipids as potential targets in cancer chemotherapy. We hypothesized that high levels of CERS6 interfere with apoptosis and render ALL cells resistant to drug treatment. *CERS6* knockdown increased T-ALL cell sensitivity to ABT-737, while *CERS6* overexpression rendered the cells resistant to ABT-737. Finally, we studied whether CERS6 binds to CD95/Fas and interferes with association of FADD to Fas, and thus inhibiting the extrinsic apoptotic pathway upon drug treatment.

## Materials and methods

### Chemicals and reagents

Sphingolipid standards includingC14:0-, C16:0-, C17:0-, C18:0-, C18:1-, C20:0-, C24:0-, C24:1-ceramide and C18:0-, C18:1-, C24:0-, C24:1-dihydroceramide were purchased from Avanti Polar Lipids (Alabaster, AL); ammonium formate and formic acid were obtained from Fisher Scientific (Pittsburg, PA); chloroform, ethyl acetate, methanol, 2-propanol, NaF, NaHCO_3_, Na_3_VO_4_, Tris-HCl, Triton X-100, pepstatin A, aprotinin, leupeptin, 200 proof ethanol, isopropanol, puromycin, dexamethasone, and anti-FLAG-M2 (1 μg/ml) antibody from Sigma-Aldrich (St. Louis, MO); ABT-737 from Cayman Chemical (Ann Arbor, MI); DTT, EDTA, NaCl, PMSF, SDS, TBE, trypsin/EDTA, Lipofectamine®, PLUS^TM^ reagent, Superscript® III first-strand synthesis system for RT-PCR from Thermo Fisher Scientific (Waltham, MA); Triton X-114 from Acros Organics (Morris, NJ); Z-IETD from R&D Systems (Minneapolis, MN); Fas ligand from GeneTex (Irvine, CA); anti-CERS6, anti-FLIP and anti-GAPDH antibodies from Santa Cruz Biotechnology (Santa Cruz, CA); anti-Fas and anti-FADD from BD Transduction Laboratories (San Jose, CA); anti-HA antibody from Roche (Indianapolis, IN); anti-caspase-3, anti-cleaved-caspase-3, anti-caspase-8 and anti-PARP from Cell Signaling Technology (Danvers, MA); *Age*1-HF, *Bam*H1-HF, *Eco*R1-HF, *Mlu*1-HF, *Pme*1 and *Sgf*1 restriction enzymes from New England Biolabs (Ipswich, MA); bovine serum albumin from Jackson ImmunoResearch Laboratories (West Grove, PA); all oligos were synthesized from Integrated DNA Technologies (Coralville, IA).

### mRNA expression of *CERS* isoforms in various cancers

The expression of messenger RNA (mRNA) in the NCI PPTP cell lines were determined in the previous study using Affymetrix U133 Microarray system^24^. The data were used to determine mRNA expression of *CERS1*-*CERS6*. CERS6 expression levels in various cancers and normal tissue samples were obtained from UCSC Xena (https://xenabrowser.net)

### Extraction of PBMCs and T lymphocytes

Human blood from normal healthy volunteers was obtained from United Blood Services (Lubbock TX). Peripheral blood mononuclear cells (PBMCs) were extracted using LSM-Lymphocyte Separation Medium (MP Biomedicals, Santa Ana, CA) as per the manufacturer’s protocol. T lymphocytes were separated from PBMCs using Dynabeads Untouched Human T Cells (Thermo Fisher Scientific, Baltics UAB) extraction kit following the manufacturer’s protocol.

### Cell culture

ALL cell lines (Supplemental Table S1) used in the study were cultured in RPMI (GE Lifesciences) supplemented with 10% heat-inactivated fetal bovine serum (FBS, Life Technologies) or Iscove’s modified Dulbecco’s medium (Life Technologies) with insulin–transferrin–selenium (Sigma-Aldrich) (10 µg/ml insulin, 5.5 µg/ml transferrin (substantially iron-free), 5 ng/ml sodium selenite), and 20% heat-inactivated FBS. All cell lines were cultured in physical hypoxia (5% CO_2_, and 5% O_2_). Cell lines were tested for and free of mycoplasma, and cell line identities were verified using short tandem repeat genotyping as compared with the original primary sample material within COGcell database: www.COGcell.org.

### T lymphocytes from primary clinical samples

Ficoll-separated mononuclear cells from peripheral blood of patients diagnosed with lymphoid malignancies were obtained from the Texas Cancer Cell Repository (TXCCR) with informed consent from all patients. T lymphocytes were separated using Dynabeads Untouched Human T Cells (Thermo fisher Scientific, Baltics UAB) extraction kit following the manufacturer’s protocol.

### Immunoblotting

Cells grown in T75 or T150 flask were washed once with ice-cold 1× phosphate-buffered saline (PBS). Cells lysis was performed on ice with modified RIPA buffer (50 mM Tris-HCl, pH 7.4, 150 mM NaCl, 1 mM EDTA, 1% Triton X-100 (Triton X-114 for co-immunoprecipitation), 1 µg/ml Leupeptin, 1 µg/ml aprotinin, 1 µg/ml pepstatin A, 1 mM PMSF, 1 mM Na_3_VO_4_ and 1 mM NaF), followed by centrifugation at 14,000 × *g* for 15 min at 4 °C. Protein concentration was determined by BCA assay (Pierce, Rockford, IL). Equal amount of protein samples were loaded and electrophoretically separated on a 4–12% sodium dodecyl sulfate–polyacrylamide gel electrophoresis (SDS-PAGE), transferred to Hybond membrane (GE Healthcare, Piscataway, NJ), blocked with 1% bovine serum albumin or 5% skim milk, immunoblotted with the indicated primary antibodies and incubated with 1:3000 horseradish peroxidase-conjugated mouse or rabbit immunoglobulin G (IgG) secondary antibodies followed by detection with enhanced chemiluminescence (GE Healthcare). The membrane was stripped and re-probed with anti-GAPDH antibody to confirm equal loading.

### Determination of ceramides by LC-MS/MS

The sphingolipids were analyzed using previously reported method^25^ with modification. Briefly, cell pellet (7.5 × 10^6^ cells) added with 50 µl of internal standard (C17:0-ceramide, 1 pM) was extracted twice with ethyl acetate/2-propanol/water (60/28/12; v/v/v). Sphingolipids were separated using gradient elution with mobile phase A (2 mM ammonium formate and 0.2% formic acid (v/v) in water) and B (1 mM ammonium formate and 0.2% formic acid (v/v) in methanol) on an Agilent 1200 series high-performance liquid chromatography (HPLC) system with Spectra C8SR column (3 µm, 150 × 3.0 mm, Peeke Scientific, Redwood, CA). Mass spectrometric detection was performed by multiple reaction monitoring (MRM) mode on a Sciex 4000 QTRAP mass spectrometer (AB Sciex, Framingham, MA) operating in positive ion mode. Analyst software 1.6.3 (AB Sciex) was used for the data acquisition as well as processing. Sphingolipid data generated from liquid chromatography-tandem mass spectrometry (LC-MS/MS) were normalized to lipid phosphate as previously described^26^.

### *CERS6* knockdown by lentiviral transduction

*CERS6* short hairpin RNA (shRNA; TRCN0000128836) in pLKO.1-puro lentiviral vector was from Dharmacon (GE Healthcare Bio-Sciences, Pittsburgh, PA). *pLKO.1-puro eGFP* shRNA sequence (Sigma-Aldrich), which targets enhanced green fluorescent protein (eGFP), was used as a non-targeting control (NT-shRNA). Knockdown of *CERS6* in CCRF-CEM or MOLT-4 cells was carried out as described under “Cell transduction” section below.

### Cloning and mutagenesis

*CERS6* complementary DNA (cDNA) was amplified by the Expand High Fidelity PCR System (Roche) using pLX304-*CERS6* (DNASU Plasmid Repository, Arizona State University) as a template and primers as specified (Supplemental Table S3). The PCR-amplified *CERS6* gene was subcloned into *Sgf*1 and *Mlu*1 sites of *pCMV6-Entry-mycDDK* (OriGene, Rockville, MD) to create *pCMV6-CERS6-mycDDK* using LigaFast^TM^ Rapid DNA Ligation System (Promega, Madison WI). *CERS6* was ligated into *Eco*R1 and *Mlu*1 sites of pLenti-HTBH-DDK^27,28^ (a kind gift from Dr. Lan Huang, UC Davis, and modified by S-JW) to be used for *CERS6* overexpression by lentiviral transduction. *FAS* cDNA was synthesized using RNA extracted from CCRF-CEM cells and amplified as mentioned above. *FAS* gene was subcloned into *Sgf*1 and *Mlu*1 sites of *pCMV6-AC-HA* (OriGene, Rockville, MD) to create *pCMV6-AC-FAS-HA*. The *FAS* mutant clones of deletion and point substitutions were generated with specific primers (Supplemental Table S3) using QuikChange Site-Directed Mutagenesis Kit (Agilent, Clara, CA). The DNA sequences of all constructs were verified by MacroGen USA (Rockville, MD) using an automated ABI-3730xl DNA Analyzer and ABI *PRISM*^®^ *BigDye*™ *Terminator* v3.0 *Ready Reaction Cycle Sequencing Kit* (Applied Biosystems, Foster City, CA). All plasmid DNAs were prepared using purification kits from Qiagen (Valencia, CA) and were endotoxin-free when used for transfection into mammalian cells.

### Cell transduction

HEK293FT (Life Technologies) cells were cultured in Dulbecco's modified Eagle's medium (Thermo Fisher Scientific) supplemented with 10% FBS, 2 mM glutamine, 100 units/ml penicillin, 100 µg/ml streptomycin sulfate and 1 mM sodium pyruvate (Life Technologies). Ten million cells were plated at cell on a 10 cm tissue culture dish and incubated at 37 °C 5% CO_2_ incubator until the cells reach 80% confluence. The HEK293FT cells were co-transfected with either lentiviral open reading frames or shRNAs along with Lenti-vpak Packaging Kit (OriGene) using the transfection reagent MegaTran 1.0 (OriGene). After 48–72 h of transfection, the virus-containing medium was collected, spun down, filtered (0.45 µm) and used for targeting into CCRF-CEM or MOLT-4 cells by infection. The virus-infected stable clones were obtained after at least 2–3 weeks of selection in 10% FBS/RPMI-1640 with 0.5 µg/ml of puromycin (Sigma-Aldrich).

### Cell transfection

HEK293FT cells at 80% confluency were transfected with recombinant DNA constructs (Fig. 6a) along with Plus reagent and Lipofectamine (both from Invitrogen, Carlsbad, CA). Cells were harvested after 48 h of transfection and samples prepared as described under “Immunoblotting” section above.

### DIMSCAN cytotoxicity assay

Cells were plated (3000 cells/well for CCRF-CEM and 4500 cells/well for MOLT-4) in a 96-well plate using 150 µl of culture medium and incubated for at least 12 h followed by 50 µl of drug treatment at the following concentrations: 0.001 to 1 µM for ABT-737 and 0.0001 to 1 µM for dexamethasone. Stock solutions for ABT-737 was prepared using dimethyl sulfoxide and dexamethasone was prepared using sterile water. DIMSCAN assay was performed for 72 h post treatment as previously described^29^.

### Apoptosis assay by flow cytometry

Apoptosis was determined using Annexin-V surface positivity by flow cytometry. CCRF-CEM or MOLT-4 cells were incubated with ABT-737, Z-IETD or combination, washed twice with PBS and subjected to Annexin-V assay using Apo Alert^TM^ Annexin-V-FITC Apoptosis kit (Takara Clontech, Mountain View, CA) as previously described^23^.

### Immunoprecipitation (IP)

The cell lines samples were prepared for IP as indicated in the “Immunoblotting” section. For purification of CERS6 (Fig. 5a), 2000 µg of protein lysates as prepared above were pulled down at 4 °C overnight with Ni-NTA His-Bind Resin (EMD-Millipore, Billerica, MA), washed 4 times with modified RIPA and then eluted with 350 mM imidazole. Further, the eluate was incubated with EZview Red Streptavidin Affinity Gel (Sigma) for 2 h, washed 4 times with modified RIPA, and digested with AcTEV Protease (Invitrogen, Carlsbad, CA) for 3 h. Immunoprecipitation of Fas was performed using 1000 µg of protein lysates incubated with 2 µg of anti-Fas antibody Rb (Proteintech, Rosemont, IL) overnight at 4 °C. Fas and associated proteins were pulled down using 50 µl of Protein G Plus Agarose beads (Santa Cruz Biotechnology, Santa Cruz, CA) and sample prepared using 4× NuPAGE lithium dodecyl sulfate loading buffer and 100 mM DTT, followed by heating at 70 °C for 10 min. For immunoprecipitation of CERS6 (Fig. 6b), 1000 µg of protein lysates were pulled down at 4 °C overnight with 50 µl EZview Red anti-FLAG-M2 affinity gels (Sigma-Aldrich), washed 4 times with modified RIPA and then eluted with an excess of 3× FLAG peptide (100 μg/ml). Immuno-complexes were resolved by 4–12% SDS-PAGE and immunoblotted with the indicated antibodies.

### Cellular fractionation

Cytoplasmic and plasma membrane fractions were isolated from 293FT whole cells using Minute™ Plasma Membrane Protein Isolation and Cell Fractionation Kit (Invent Biotechologies, Plymouth, MN) as specified by the manufacturer's protocol.

### FAS surface expression

FAS expression levels were determined in CERS6 knockdown and overexpressing CCRF-CEM cells by flow cytometry using CD95-FITC and FITC Mouse Anti-human IgG (both from BD Biosciences, San Jose, CA) as described previously^30^. Briefly, cells at logarithmic growth phase were washed twice with PBS, and resuspended in 100 μl of binding buffer (BD Biosciences) at a final concentration of 0.5 × 10^6^ cells per 50 μl. Either anti-human IgG-FITC or CD95-FITC (20 μl per sample) was added to the cell suspension, and the mixure was indubated for 10 min at room temperature. The cells were washed and resuspended in 390 μl of binding buffer. The cells were analyzed by flow cytometry (BD LSR II operated by FACS DIVA) with band-pass filters of 525 ± 25 mm.

### Statistical analysis

Assessment of significance were performed using Student’s *t*-test and test results were considered significant at *p* < 0.05. Data were plotted and analyzed using GraphPad Prism 6 and SigmaPlot v11.

## Results

### CERS6 is highly expressed in T-ALL cells in comparison to normal cells

The mRNA expression of six CERS isoforms was analyzed in the NCI PPTP (National Cancer Institute Pediatric Preclinical Testing Program) panel of 23 cell lines^29^. *CERS2*, *CERS5* and *CERS6* expression levels were significantly higher than *CERS1*, *CERS3* and *CERS4* (8.7 ± 0.4, 7.9 ± 0.3 and 8.6 ±1.0 vs 5.0 ± 0.3, 3.2 ± 0.1 and 4.7 ± 0.7, *p* < 0.0001, Fig. 1b). Moreover, the relative expression was consistent (CV for *CERS1*: 5.6%, *CERS2*: 4.2%, *CERS3*: 2.2%, *CERS4*: 14.1%, *CERS5*: 3.9% and *CERS6*: 12.1%) in all the 23 cell lines analyzed, but different from the tissue distribution in normal cells^11^. *CERS2* has been reported to be the most abundant enzyme with highest expression in most tissues, while mRNA expression of *CERS6* is low in major tissues^11^. Here, we observed that the levels of *CERS6*, responsible for the generation of C_16_-Cer, were as high in cancer cells and similar to the levels of *CERS2* (8.7 ± 0.4) among all cell lines tested. CERS6 was also found to be overexpressed in several other adult cancers when compared to normal tissue samples (Supplemental Fig. S1).

Next, we compared CERS6 protein levels in 10 leukemia cell lines (Supplemental Table S1)^31^ to PBMCs and T lymphocytes isolated from blood of healthy human volunteers, representing normal cells. CERS6 levels were significantly higher in ALL cells compared to PBMCs or T lymphocytes (Fig. 1c). To verify the functionality of CERS6, the levels of C_16_-Cer, the product of CERS6, were quantitated in ALL and normal cells by LC/MS/MS. As anticipated, C_16_-Cer levels were significantly higher in ALL cell lines in comparison to PBMCs or T lymphocytes (0.54 ± 0.09 vs 0.28 ± 0.04, *p* < 0.001 or 0.36 ± 0.01, *p* < 0.001, Fig. 1d). To investigate whether CERS6 levels are elevated in primary T-lymphoid malignancies, we employed clinical samples from four lymphoid malignancy patients (Supplemental Table S2) and found that the levels of CERS6 were higher in T lymphocytes isolated from the four patient samples in comparison to normal T lymphocytes (Fig. 1e).

### CERS6 alters sensitivity of ALL cells to ABT-737, a pan-BCL-2 family of protein inhibitor

**Fig. 2:**
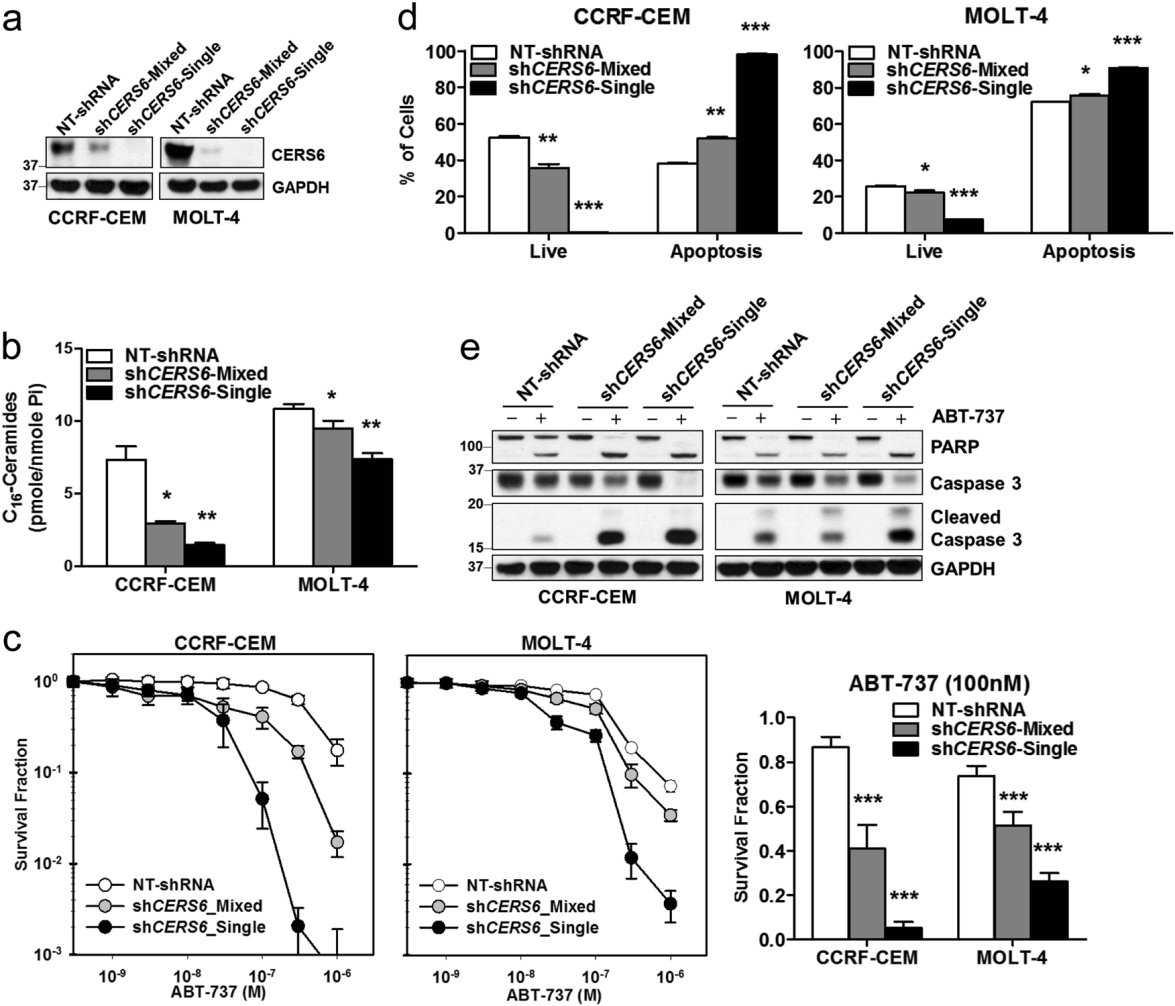
ALL cells are sensitized to ABT-737 upon *CERS6* knockdown. **a** CERS6 protein levels in CCRF-CEM and MOLT-4 cells after stably knocking down *CERS6* using shRNA (NT-shRNA non-targeted shRNA, sh*CERS6*-Mixed shRNA against *CERS6* and selected with puromycin, sh*CERS6*-Single shRNA against *CERS6* and selected with puromycin followed by repopulation from a single cell). GAPDH was used as a loading control. **b** C_16_-Ceramide levels (pmole/nmole of inorganic phosphate) in ALL cells after *CERS6* knockdown. Ceramide levels were quantitated by HPLC/MS/MS (1.5 ± 0.1 vs 7.3 ± 0.9 pmole/nmole Pi for CCRF-CEM and 7.4 ± 0.4 vs 10.9 ± 0.3 pmole/nmole Pi for MOLT-4; *n* = 3). **c** Knockdown of *CERS6* in CCRF-CEM and MOLT-4 sensitized the cells to ABT-737. Dose response curves showing concentration of ABT-737 on *x*-axis and survival fraction on *y*-axis on a log_10_ scale. Bar graphs depict survival fractions at 100 nM of ABT-737 in both cell lines (5.2 ± 2.7% vs 86.6 ± 4.6% survival at 100 nM ABT-737 for CCRF-CEM and 26.1 ± 3.8% vs 73.6 ± 4.6% survival at 100 nM ABT-737 for MOLT-4; *n* = 6). **d** Annexin-V apoptosis assay by flow cytometry showing the percentage of live and apoptotic cells upon ABT-737 treatment (1 µM for 16 h) in CCRF-CEM or MOLT-4 cells with *CERS6* knockdown in comparison to cells transduced with NT-shRNA (98.0 ± 0.4% vs 38.0 ± 0.4% for CCRF-CEM and 90.8 ± 0.3% vs 72.1 ± 0.3% for MOLT-4; *n* = 3). **e** *CERS6* knockdown in CCRF-CEM or MOLT-4 cells show higher levels of cleaved PARP and cleaved caspase-3 on ABT-737 treatment (1 µM for 16 h). GAPDH was used as a loading control * *p* < 0.05, ** *p* < 0.01, *** *p* < 0.001

To determine whether higher levels of CERS6 in ALL contributes to resistance in cytotoxicity of chemotherapeutic drugs, we carried out loss-of-function (knockdown of *CERS6*) and gain-of-function (*CERS6* overexpression) experiments in ALL cells. *CERS6* was stably knocked down using shRNA by lentiviral transduction in two T-ALL cell lines, CCRF-CEM and MOLT-4. The decrease in CERS6 levels after *CERS6* knockdown was confirmed by immunoblotting (Fig. 2a). C_16_-Cer levels were significantly reduced in *CERS6* knockdown cells compared with cells transduced with non-targeted shRNA (1.5 ± 0.1 vs 7.3 ± 0.9 pmole/nmole Pi, *p* < 0.01 for CCRF-CEM and 7.4 ± 0.4 vs 10.9 ± 0.3 pmole/nmole Pi, *p* < 0.01 for MOLT-4, Fig. 2b), demonstrating that the CERS6 activity was also reduced with the decrease in CERS6 levels in both cell lines. Then, the effect of CERS6 knockdown on the cytotoxicity of ABT-737, a pan-BCL-2 inhibitor, was evaluated. Both CCRF-CEM and MOLT-4 cells with *CERS6* knockdown displayed a significant increase in cytotoxicity upon ABT-737 treatment (5.2 ± 2.7% vs 86.6 ± 4.6% survival at 100 nM ABT-737, *p* < 0.001 for CCRF-CEM and 26.1 ± 3.8% vs 73.6 ± 4.6% survival at 100 nM ABT-737, *p* < 0.001 for MOLT-4, Fig. 2c). In both cell lines with *CERS6* knockdown, the percentage of apoptotic cells in response to ABT-737 treatment was significantly higher compared with the cells transduced with non-targeted shRNA (98.0 ± 0.4% vs 38.0 ± 0.4%, *p* < 0.0001 for CCRF-CEM and 90.8 ± 0.3% vs 72.1 ± 0.3%, *p* < 0.0001 for MOLT-4, Fig. 2d and Supplemental Fig. S2). Immunoblotting showed that cleaved poly (ADP-ribose) polymerase (PARP) and cleaved caspase-3, markers for apoptotic cell death, were increased in both cell lines with *CERS6* knockdown upon ABT-737 treatment in comparison to cells transduced with non-targeted shRNA (Fig. 2e).

**Fig. 3:**
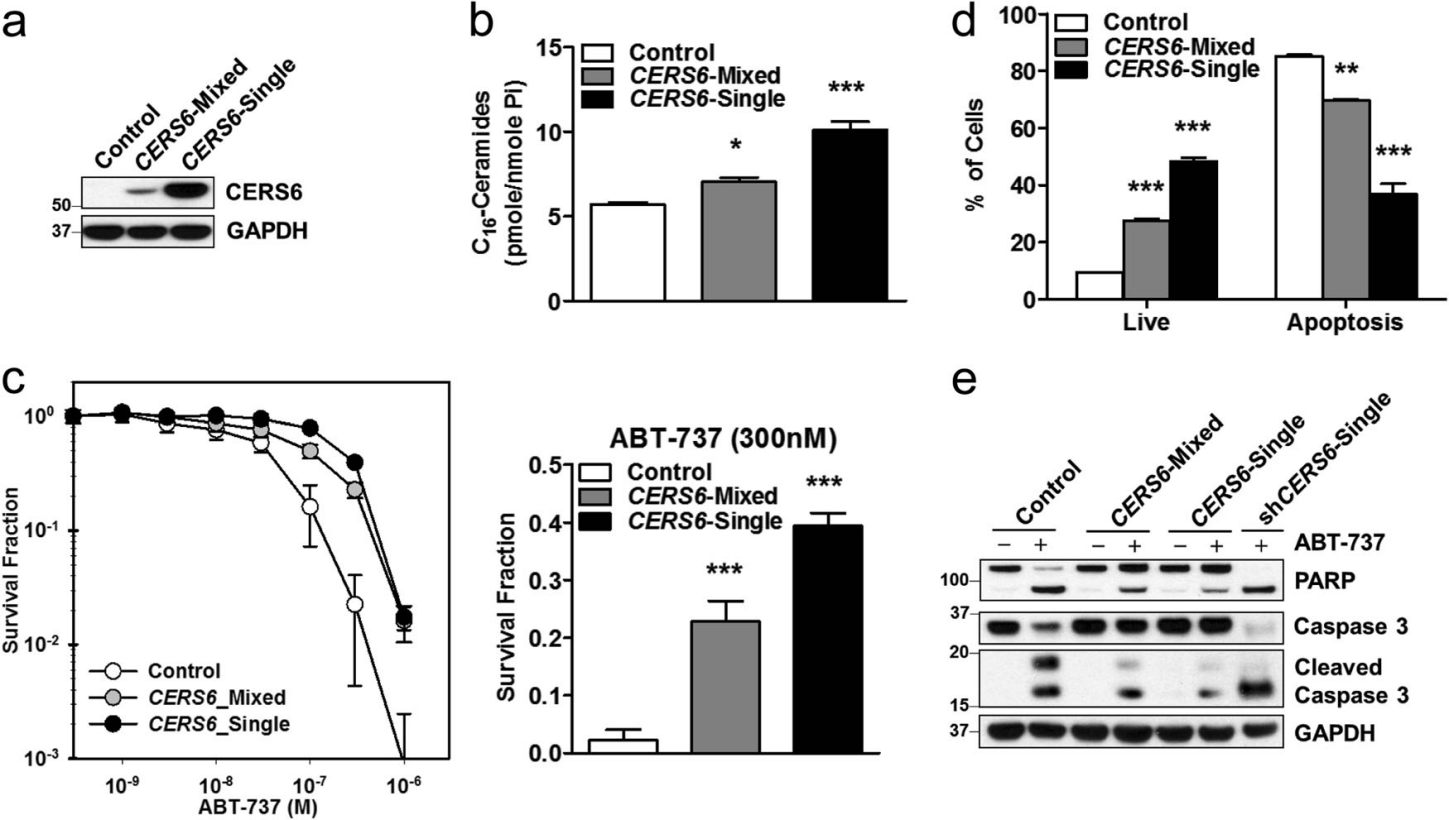
ALL cells overexpressing *CERS6* show resistance to ABT-737. **a** CERS6 levels in CCRF-CEM cells upon *CERS6* overexpression (Control cells transduced with empty vector, *CERS6*-Mixed *CERS6* overexpressing cells and selected with puromycin, *CERS6*-Single *CERS6* overexpressing cells and selected with puromycin followed by repopulation from a single cell; 10.1 ± 0.5 vs 5.7 ± 0.1 pmole/nmole Pi; *n* = 3). **b** C_16_-Cer levels (pmole/nmole of inorganic phosphate) in CCRF-CEM cells after *CERS6* overexpression. Ceramide levels were quantitated by HPLC/MS/MS. **c** *CERS6* overexpression in CCRF-CEM cells rendered the cells resistant to ABT-737. Dose response curves showing concentration of ABT-737 on *x*-axis and survival fraction on *y*-axis on a log_10_ scale. Bar graphs depict survival fractions at 300 nM of ABT-737.(39.5 ± 2.2% vs 2.3 ± 1.8% survival at 300 nM ABT-737; *n* = 6) (**d**) Annexin-V apoptosis assay by flow cytometry showing the percentage of live and apoptotic cells upon ABT-737 treatment (2 µM for 16 h) in *CERS6* overexpressing CCRF-CEM cells in comparison to cells transduced with empty vector (36.8 ± 3.8% vs 85.2 ± 0.6%; *n* = 3). **e** *CERS6* overexpressing CCRF-CEM cells show lower levels of cleaved PARP and cleaved Caspase-3 on ABT-737 treatment (2 µM for 16 h) in comparison to control cells. The far right lane (sh*CERS6*-Single) is a sample from ABT-737-treated *CERS6* knockdown CCRF-CEM cells (from Fig. 2e) and represents positive control for cleaved PARP and cleaved caspase-3. GAPDH was used as a loading control * p < 0.05, ** p < 0.01, *** p < 0.001

Based on the expression of CERS6 in ALL cells, CCRF-CEM cells with relatively low CERS6 expression was selected for exogenous expression of CERS6. After stably expressing CERS6 in CCRF-CEM cells, the increase in CERS6 protein was confirmed in comparison to cells transduced with control vector (Fig. 3a). C_16_-Cer levels in CCRF-CEM cells with exogenous CERS6 expression were significantly higher relative to its control cells (10.1 ± 0.5 vs 5.7 ± 0.1 pmole/nmole Pi, *p* < 0.001, Fig. 3b). Increase in CERS6 levels rendered CCRF-CEM cells resistant to ABT-737 in comparison to control cells (39.5 ± 2.2% vs 2.3 ± 1.8% survival at 300 nM ABT-737, *p* < 0.001, Fig. 3c). The percentage of apoptotic cells were significantly lower (36.8 ± 3.8% vs 85.2 ± 0.6%, *p* < 0.01, Fig. 3d), and cleaved PARP and caspase-3 were less (Fig. 3e) in CCRF-CEM cells overexpressing *CERS6* in comparison to cells transduced with control vector when treated with ABT-737.

### CERS6-mediated resistance to ABT-737 occurs via the extrinsic pathway of apoptosis

**Fig. 4:**
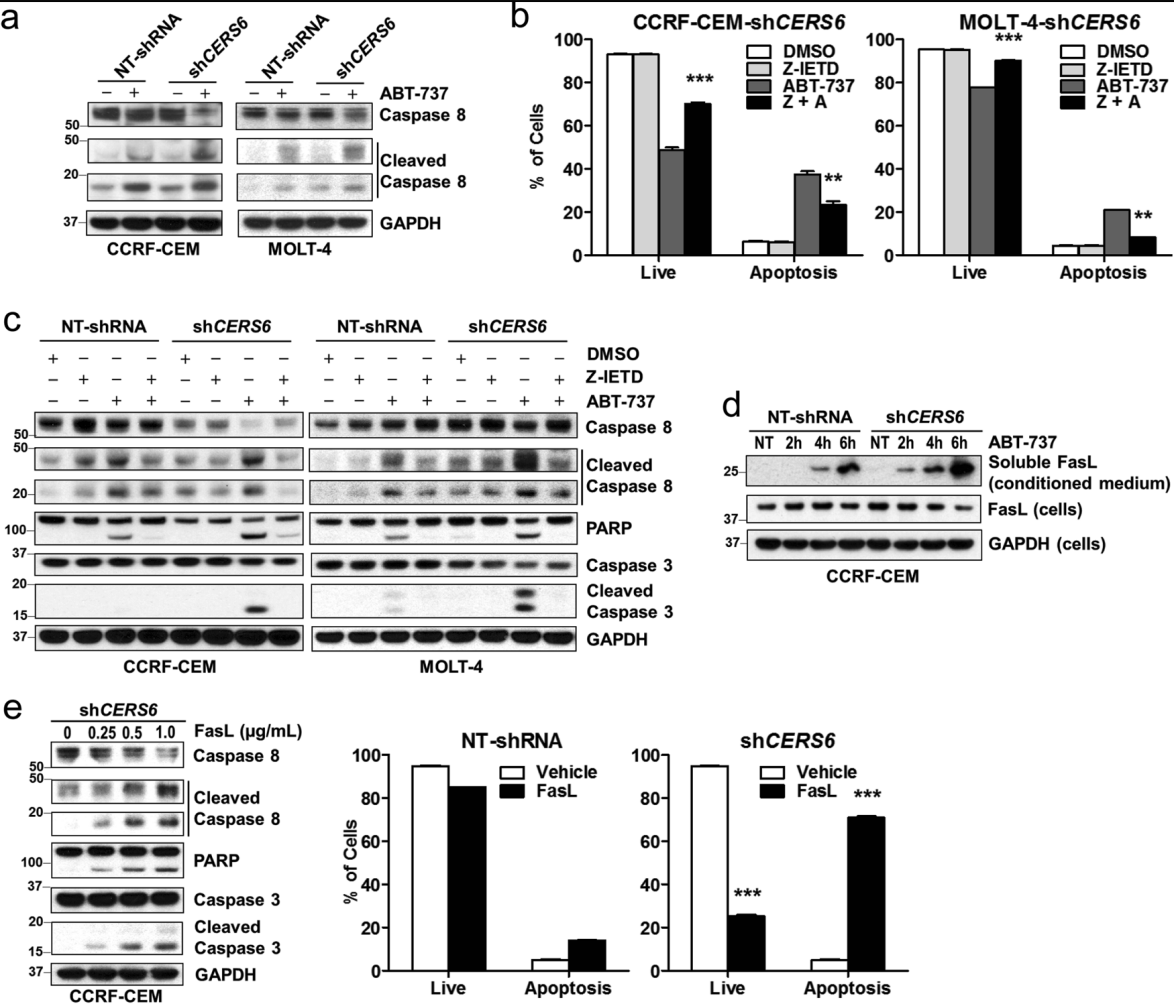
CERS6 alters ALL cells sensitivity to ABT-737 via the extrinsic pathway of apoptosis which can be overcome by a caspase-8 inhibitor. **a** Higher caspase-8 activity seen with CCRF-CEM and MOLT-4 CERS6 knockdown cells in comparison to cells transduced with non-targeted shRNA upon ABT-737 treatment. GAPDH was used as a loading control. **b** Annexin-V apoptosis assay by flow cytometry showing the percentage of apoptotic cells in ABT-737-treated CERS6 knockdown cells (CCRF-CEM and MOLT-4), with or without caspase-8 inhibitor, Z-IETD (23.4 ± 1.7% vs 37.5 ± 1.5% for CCRF-CEM and 8.2 ± 0.1% vs 20.8 ± 0.3% for MOLT-4; *n* = 3). **c** ABT-737-treated CERS6 knockdown cells show decreased levels of cleaved Caspase-8, cleaved PARP and cleaved caspase-3 on addition of Z-IETD in comparison to ABT-737-treated CERS6 knockdown cells without Z-IETD. **d** Levels of soluble FasL released in cultured medium upon treatment of ABT-737 in CERS6 knockdown cells compared to cells transduced with non-targeted shRNA. **e** Left: Caspase-8 activity was increased in a dose-dependent manner upon treatment of varying concentrations of Fas ligand (FasL) in CCRF-CEM cells with CERS6 knockdown. GAPDH was used as a loading control. **e** Right: Annexin-V apoptosis assay by flow cytometry showing increase in apoptotic cells in CCRF-CEM cells with CERS6 knockdown in comparison to cells transduced with NT-shRNA upon treatment of 1000 ng/ml of FasL (70.9 ± 0.6% vs 14.1 ± 0.1%; *n* = 3) ** p < 0.01, *** p < 0.001

ABT-737-inducing apoptosis via mitochondrial (intrinsic) pathway of apoptosis is well documented. However, the extent of changes in anti- and pro-apoptotic BCL-2 family of proteins in ALL cells with *CERS6* knockdown or exogenous expression was not consistent with the differences in cytotoxicity of ABT-737 (Supplemental Fig. S3a). Ceramides in plasma membranes are reported to form “ceramide-enriched platforms” which aid in clustering of death receptors in the extrinsic pathway of apoptosis^32^. Thus, the effect of CERS6 modulation in extrinsic apoptotic pathway in response to ABT-737 treatment was investigated. The involvement of extrinsic apoptotic pathway was assessed by measuring the levels of cleaved caspase-8 in CCRF-CEM and MOLT-4 cells with *CERS6* knockdown upon treatment of ABT-737. Higher levels of cleaved caspase-8 were observed in both cell lines with *CERS6* knockdown compared with their respective controls (Fig. 4a), demonstrating that CERS6 is associated with ABT-737 resistance via the extrinsic pathway of apoptosis. Then, CCRF-CEM and MOLT-4 with *CERS6* knockdown were treated with Z-IETD, a caspase-8 inhibitor, 1 h prior to ABT-737 treatment. The percentage of apoptotic cells was significantly decreased in cells with *CERS6* knockdown pretreated with Z-IETD in response to ABT-737 in comparison to cells without Z-IETD pretreatment (23.4 ± 1.7% vs 37.5 ± 1.5%, *p* < 0.01 for CCRF-CEM and 8.2 ± 0.1% vs 20.8 ± 0.3%, *p* < 0.01 for MOLT-4, Fig. 4b). Cleaved caspase-8, PARP and caspase-3 were reduced by the addition of Z-IETD in *CERS6* knockdown cells treated with ABT-737 compared with cells without the caspase-8 inhibitor (Fig. 4c). We measured changes in soluble Fas ligand (FasL) in culture medium upon treatment of ABT-737 and found that ABT-737 induced the release of soluble FasL into the medium and that FasL release by ABT-737 was earlier and greater in CERS6 knockdown cells relative to its empty vector control (Fig. 4d). Further, when CCRF-CEM cells were treated with soluble FasL, cells with *CERS6* knockdown showed significantly higher percentage of apoptotic cells compared with cells transduced with non-targeted shRNA (70.9 ± 0.6% vs 14.1 ± 0.1%, *p* < 0.0001, Fig. 4e). CCRF-CEM cells with *CERS6* knockdown were sensitized to dexamethasone, part of ALL standard treatment, in comparison to control cells (32.5 ± 11.9% vs 2.2 ± 1.1% survival at 100 nM, *p* < 0.001, Supplemental Figs. S3b, c).

### CERS6 binds to CD95/Fas and interferes with FADD assembly to Fas in the extrinsic pathway of apoptosis

**Fig. 5:**
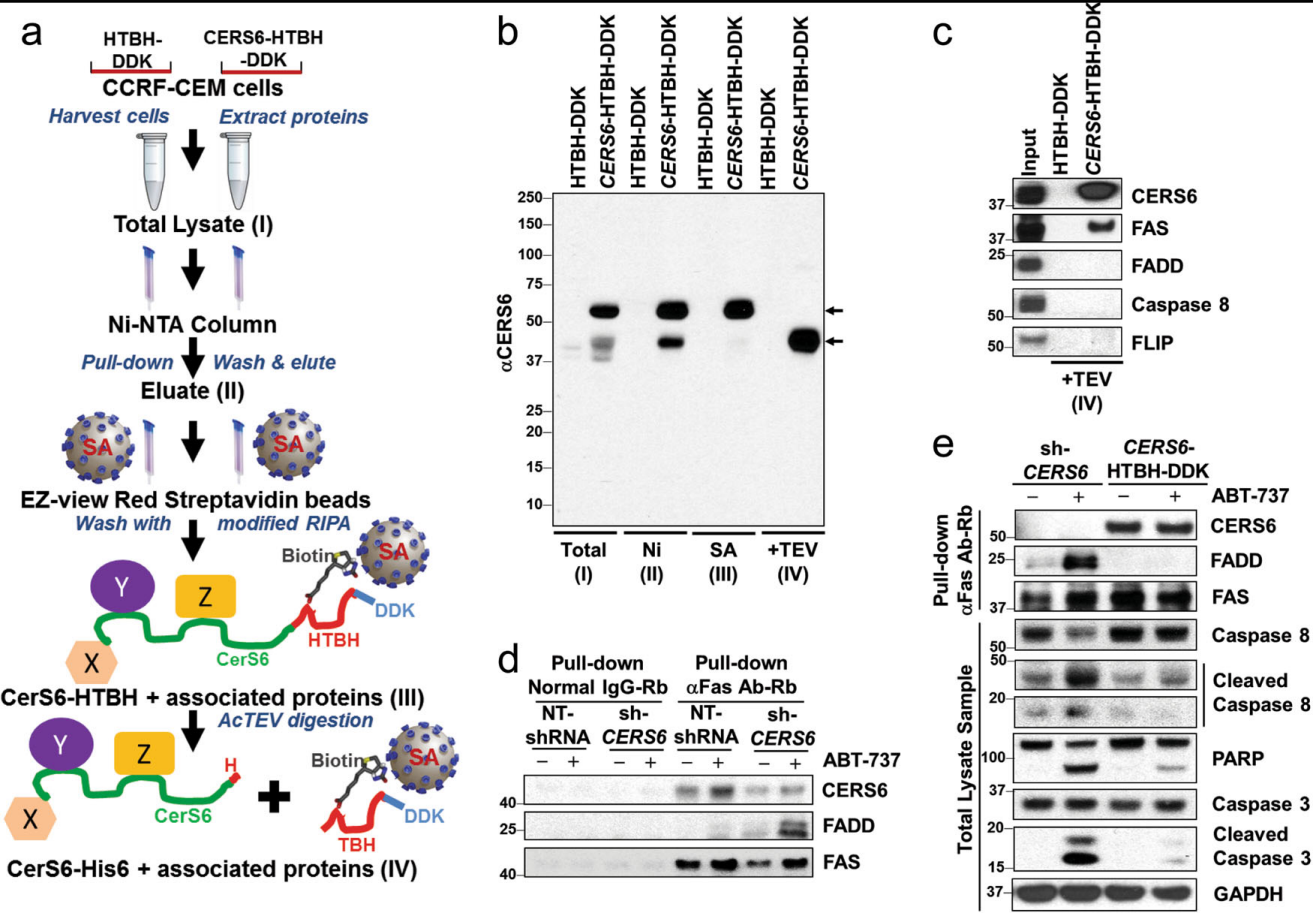
CERS6 binds to CD95/Fas and interferes with FADD association to Fas in the extrinsic pathway of apoptosis. **a** Schematic diagram listing the steps involved in the purification of CERS6 along with associated proteins from *CERS6* overexpressing CCRF-CEM cells. HTBK-DDK (His6-TEV-Biotinylated region-His6-DDK) is a tag placed at the C-terminal site on CERS6 overexpressing vector. X, Y and Z represent unknown proteins associated with CERS6. **b** Confirmation of CERS6 protein expression at various purification steps. (I) total lysate sample; (II) pull-down with Ni-NTA beads; (III) pull-down with streptavidin beads; and (IV) sample after AcTEV digestion (the TBH-DDK part of the tag is cleaved off). **c** Detection of Fas as a binding partner of CERS6. Other proteins involved in the DISC assembly of the extrinsic pathway did not bind to CERS6. **d** Higher FADD–Fas association in *CERS6* knockdown cells treated with ABT-737. CERS6 and FADD were detected in Fas pull-down samples. Normal IgG was used as a control. **e** FADD–Fas association is decreased in ABT-737-treated *CERS6* overexpressing CCRF-CEM cells in comparison to cells with *CERS6* knockdown. Cleaved caspase-8, cleaved PARP and cleaved caspase-3 levels were compared between *CERS6* knockdown and overexpressing cells treated with ABT-737

After showing that CERS6 interferes with the extrinsic pathway of apoptosis, we sought to determine the mechanism by which caspase-8 activation is attenuated by CERS6. We wanted to see if CERS6 interacted with any of the key players involved in the extrinsic pathway of apoptosis, namely Fas death receptor, FADD (Fas-associated protein with death domain), or caspase-8. Co-immunoprecipitation of CERS6 in CERS6 overexpressing CCRF-CEM cells using a two-step purification protocol (Fig. 5a) was conducted. The efficiency of the pull-down was confirmed by detecting CERS6 at different stages of purification (Fig. 5b). The results showed that CERS6 binds to Fas death receptor, also known as CD95, when the final pull-down sample was probed with Fas antibody (Fig. 5c). Fas ligand binds to Fas death receptor to form the death-inducing signaling complex (DISC) composed of Fas, FADD and pro-caspase-8^33^ which initiates caspase-8 cleavage, further transducing a downstream signaling cascade of caspase activation resulting in apoptosis. Then, Fas was pulled down in ABT-737-treated CCRF-CEM cells to assess the effect of CERS6 on FADD association with Fas. We found that higher levels of FADD were associated with Fas in cells with *CERS6* knockdown as compared to control cells on treatment with ABT-737 (Fig. 5d). We also found a decrease in FADD–Fas association in CCRF-CEM cells overexpressing *CERS6* on ABT-737 treatment in comparison to ABT-737-treated CCRF-CEM *CERS6* knocked down cells (Fig. 5e). Together, these results suggest that CERS6 binds to Fas and inhibits its assembly with FADD leading to decreased apoptosis via the extrinsic pathway.

### CERS6 binds to intracellular domains of CD95/Fas

**Fig. 6:**
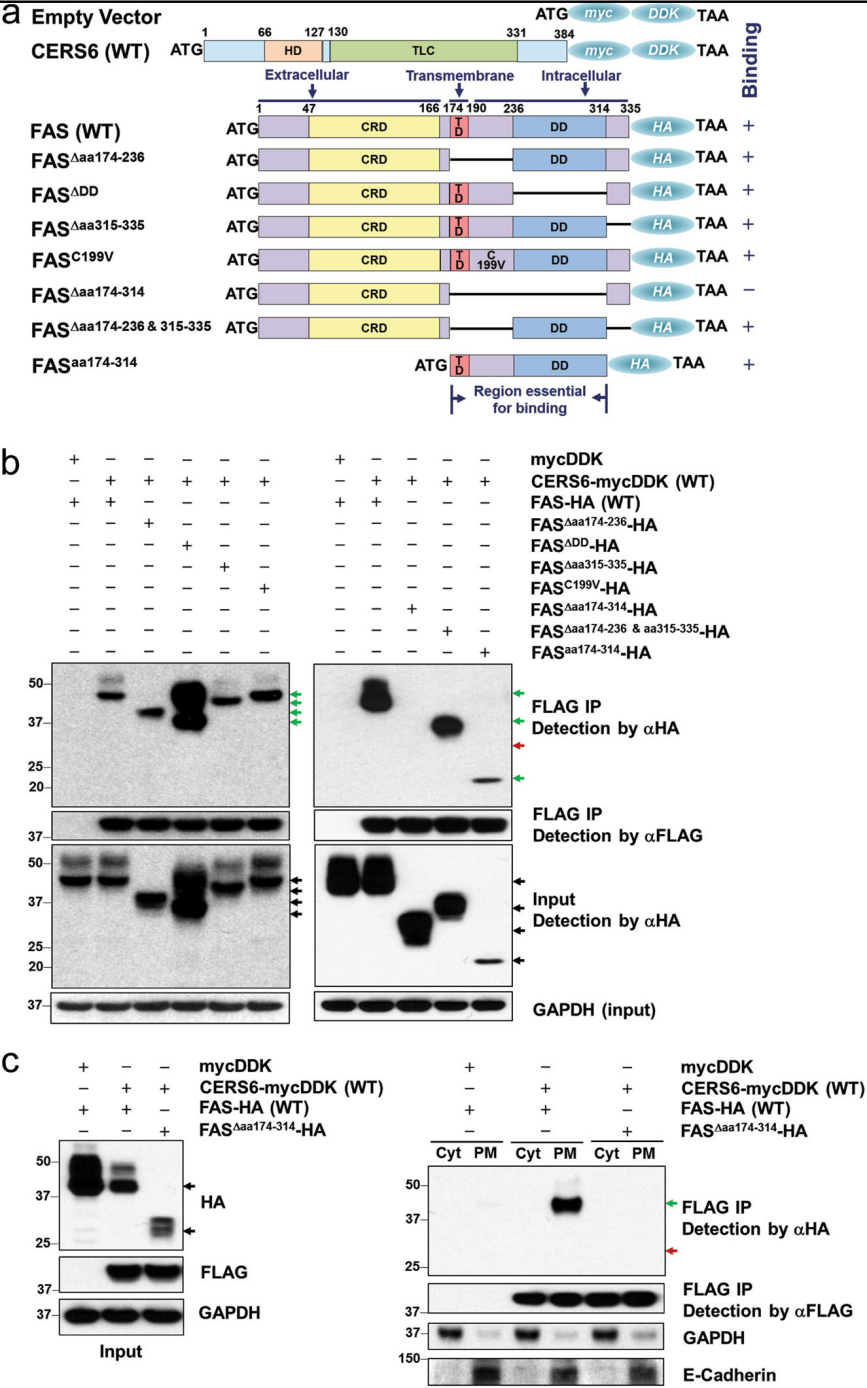
CERS6 binds to intracellular domains of CD95/Fas. **a** Constructs (pCMV6-mycDDK or pCMV6-AC-HA) encoding a human wild-type *CERS6* tagged with mycDDK at COOH terminus, *FAS* full-length and *FAS* mutants tagged with HA epitopes. HD homeobox (DNA binding) domain, TLC TRAM/LAG/CLN8 homology domain, CRD cysteine-rich domain, TD transmembrane domain, DD death domain. **b** Co-immunoprecipitation of full-length CERS6 and Fas and Fas mutants to detect direct binding. **c** Co-immunoprecipitation of full-length CERS6 and Fas/Fas mutants in cellular fractions (Cyt cytosolic fraction that includes ER and other organelles, PM plasma membrane). GAPDH (right) is used as a marker for cytosolic fraction while E-cadherin is the marker for plasma membrane

**Fig. 7:**
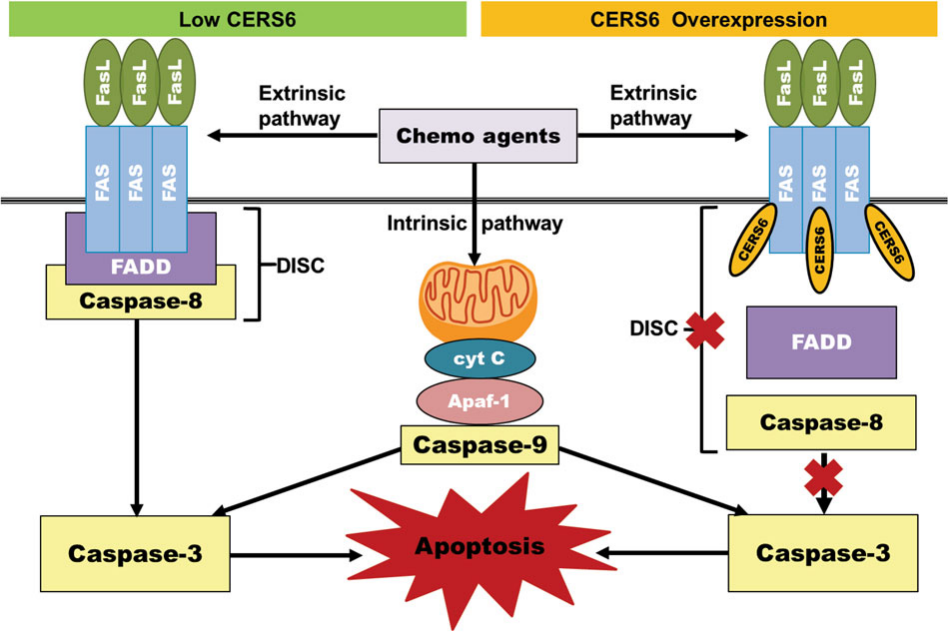
Mechanism of CERS6-induced resistance. Proposed mechanism of CERS6-mediated resistance to chemotherapy via extrinsic pathway of apoptosis. Left: FasL binds to Fas and recruits FADD to form DISC complex which activates caspase-8 to induce apoptosis upon drug treatment. Right: CERS6 bound to Fas inhibits the formation of DISC complex and inhibits extrinsic apoptosis, and thus it inhibits downstream activation of apoptosis upon drug treatment, resulting in resistance

In order to determine the binding site of CERS6 on Fas, CERS6 and Fas were exogenously expressed in HEK293FT cells using two distinct tags, CERS6 with mycDDK and Fas with HA tag. To determine if any particular domain was critical for CERS6-Fas binding, deletion mutants of Fas intracellular region were constructed as shown in Fig. 6a. The binding was anticipated to occur in the death domain, the region where FADD binds to Fas to form DISC^34^, but, the deletion of the death domain only, did not affect CERS6 binding to Fas (Fig. 6b, left). Cysteine 199 residue adjacent to the transmembrane domain of Fas has been reported to be critical for the ability of Fas to trigger apoptosis^35^. The binding persisted even with the C199V mutation on Fas, suggesting that this residue is not critical for CERS6 binding to Fas. The binding of CERS6 to each of the deletion mutants in the intracellular region of Fas indicates that CERS6 binding to Fas involves multiple sites of Fas. To confirm this, deletion mutants (∆174–314 and ∆174–236 and 315–335, Fig. 6a) of the Fas intracellular region were constructed. The binding was lost with the deletion of amino acids 174 to 314, the region comprising the transmembrane domain to death domain, while the deletion of the entire cytoplasmic region except for the death domain did not affect the binding, suggesting that the binding occurs at more than one region from amino acids 174 to 314 (Fig. 6b, right). Further, we also confirmed that the binding of CERS6 and FAS mainly occurs in in plasma membrane relative to cytoplasmic fraction (which includes endoplasmic reticulum (ER) and other organelles) even with exogenous expression of CERS6 in cytoplasm (Fig. 6c). To conclude, our data show that CERS6 binds to Fas and inhibits the formation of DISC thereby blocking apoptosis upon treatment of drugs acting via extrinsic pathway of apoptosis (Fig. 7).

## Discussion

Sphingolipid profiles are frequently altered between cancer cells and normal cells, which may be an indication of prevailing mechanisms used by tumor cells to overcome stressors. Here we report (1) that ceramide synthase-6 (CERS6), an enzyme responsible for the generation of C16 ceramides, is overexpressed in T-cell ALL cells compared with T lymphocytes and PBMCs; (2) that genetic modification of *CERS6* in T-cell ALL cell lines resulted in significant changes in sensitivity to chemotherapeutic drugs; and (3) that CERS6 binds to Fas and renders resistance to chemotherapy via the extrinsic apoptotic pathway by interfering with the Fas–FADD association in ALL cells.

Several studies have examined how the metabolism of bioactive sphingolipids is reconfigured in cancer and whether modulating the balance between pro-apoptotic and pro-survival sphingolipids can overcome resistance to anticancer drugs^12^. For instance, treatment of chemo-resistant chronic myeloid leukemia cells with a BCR-ABL tyrosine kinase inhibitor demonstrated that increase in ceramide synthases preceded cell death^36^. Treatment of HL-60/VCR, the multidrug-resistant variant of HL-60 leukemia cells, with curcumin induced an increase in ceramide generation and accumulation, leading to apoptosis^37^. However, a majority of these studies consider ceramide species and corresponding ceramide synthases to be pro-apoptotic, without species-specific differences in activity or the mechanisms of inducing or interfering apoptosis in cancer cells in response to chemotherapy. Moreover, no studies have addressed the biological function of ceramide synthases apart from acylation of sphinganine to form ceramides. Our study, for the first time, elucidates the non-enzymatic role of CERS6, wherein its binding to Fas blocks the activation of the extrinsic apoptotic pathway in ALL cells upon treatment of anticancer drugs.

Our observation on higher levels of CERS6 and C_16_-Cer in T-ALL cell lines is consistent with a few other cancers as reported in the literature. C_16_-Cer levels were significantly higher in breast cancer relative to normal tissues^38^, while CERS6 mRNA levels were found to be expressively increased in ER-positive tumors in comparison to ER-negative tumors^39^. In non-small cell lung cancer, *CERS6* levels were markedly higher in comparison to controls and was found to be associated with poor prognosis and increased metastasis^40^. C_16_-Cer levels were significantly increased in HNSCC which correlated with higher mRNA expression of *CERS6*, when 12 pairs of HNSCC tumors and normal tissues were compared^41^. In our study, we have shown higher expression of CERS6 in T-ALL in comparison to normal cells is associated with T-ALL drug resistance.

Previously, it has been shown that knockdown of *CERS6* in HNSCC cells induced stress-mediated apoptosis which could be reversed by overexpressing *CERS6* in these cells^17^. We knocked down *CERS6* as well as overexpressed it in ALL cell lines and showed that these genetic alterations changed the sensitivity of ALL cells to chemotherapy. Our studies further demonstrated that CERS6-altered ALL sensitivity to ABT-737 occurs via the extrinsic pathway, by using a caspase-8 inhibitor. Although ABT-737 is anticipated to primarily act via the mitochondrial pathway of apoptosis, it has also been reported to act through the extrinsic pathway, where it enhances TRAIL-mediated cytotoxicity in renal, prostate and lung cancer cells by upregulating TRAIL receptor, death receptor 5^42^. Our study suggests the expression of CERS6 as the mediator in inducing resistance to chemotherapy acting via extrinsic pathway of apoptosis.

We have demonstrated that there is clear processing of caspase-8 upon ABT-737 treatment, in order to show the involvement of extrinsic apoptotic pathway. However, caspase cleavage during late-stage apoptosis does not faithfully indicate one pathway or another^43^. To further confirm the involvement of extrinsic pathway, we conducted experiments by treating the cells with FasL, which is exclusive to the extrinsic pathway of apoptosis and showed that caspase-8 is activated in CERS6 knockdown cells (Fig. 4e). In addition, we have studied the effect of knockdown or overexpression of CERS6 on the cell surface expression of FAS. We conducted flow cytometric analysis of FAS cell surface expression in CERS6 knockdown and exogenous expressed cells. Interestingly, CERS6 knockdown resulted in increased FAS cell surface expression (Supplemental Fig. S4). However, CERS6 overexpression did not affect the expression of FAS. The mechanism of FAS increase with the knockdown of CERS6 warrants further investigation in future studies.

Finally, we show that CERS6 binds to Fas and interferes with Fas–FADD association to form DISC complex which activates caspase-8 in the extrinsic pathway of apoptosis. Fas has been reported to interact with a palmitoyl transferase, DHHC7 (aspartate-histidine-histidine-cysteine family of acyl transferases) which stabilizes Fas by palmitoylation at C199^35^. Being an acyl transferase, CERS6 was thought to interfere with binding of DHHC7 thereby destabilizing Fas and its ability to cause apoptosis. Binding of CERS6 to Fas persisted with C199V mutation, suggesting that C199 is not critical for CERS6 binding. BCL2L13 and SIRT3 are two proteins reported to be interacting with CERS6, where the former is shown to inhibit the activity of CERS6^44^, while the latter deacetylates CERS6 to cause an increase in its activity^45^. These interactions affect the activity of CERS6, but there are no reports showing that CERS6 affects the function of other proteins. Here, we show that CERS6 affects the function of Fas by directly interfering with its assembly with FADD.

In summary, our study shows that CERS6 plays an important role in ALL resistance to chemotherapy by interfering with the Fas–FADD assembly in the extrinsic pathway of apoptosis. CERS6 may serve as a biomarker to stratify ALL patients to determine whether they were likely to respond to drugs acting via the extrinsic pathway of apoptosis. As cancer cells continue to develop resistance to chemotherapeutic drugs and evolve new mechanisms to evade apoptosis, future studies on determining CERS6 as a biomarker for drug resistance in cancer are warranted.

## Electronic supplementary material

Supplemental Tables

Supplemental Figure Legends

Supplemental Figure 1

Supplemental Figure 2

Supplemental Figure 3

Supplemental Figure 4
