# Supplementary material for: Ceramide synthase-6 confers resistance to chemotherapy by binding to CD95/Fas in T-cell acute lymphoblastic leukemia
Source: Cell Death Dis. 2018 Sep 11;9(9):925. doi: 10.1038/s41419-018-0964-4 (PMC6133972; doi:10.1038/s41419-018-0964-4)
Supplement: Supplementary file 1 — Supplemental Tables [file 41419_2018_964_MOESM1_ESM.docx]

**Supplemental Table S1:** Cell lines used in this study^27^

| **Cell Line** | **Phase of Therapy** | **Age (yrs)** | **Sex** | **Diagnosis** | **Sample type** | **Source** | **STR** | **References** | **Year*** |
| --- | --- | --- | --- | --- | --- | --- | --- | --- | --- |
| COG-LY-357h | Dx | NA | NA | T-cell lymphoblastic lymphoma | Bone marrow | COGcell | V | unpublished | 2007 |
| COG-LL-434h | Dx | NA | NA | T-cell ALL | Bone marrow | COGcell | V | unpublished | 2012 |
| COG-LL-329h | Dx | NA | M | T-cell ALL | Bone marrow | COGcell | V | Leuk Res. 342-349, 2012 | 2007 |
| COG-LL-332h | PD | 10 | M | T-cell ALL | Bone marrow | COGcell | V | Exp.Cell Res. 78–89, 2015 | 2006 |
| CCRF-CEM | PD | 4 | F | T-cell ALL | Peripheral blood | ATCC | V^c^ | Cancer 522-529, 1965 | 1993 |
| TX-LY-172h | PD | 8 | NA | T-cell ALL | Bone marrow | TXCCR | V | unpublished | 2013 |
| MOLT-3 | PD | 19 | M | T-cell ALL | Peripheral blood | ATCC | V^c^ | Blut. 99-103, 1981 | 1984 |
| MOLT-4 | PD | 19 | M | T-cell ALL | NA | ATCC | V^c^ | Blut. 99-103, 1981 | 1985 |
| COG-LL-317h | PD | 2 | M | T-cell ALL | Bone marrow | COGcell | V | Exp.Cell Res. 78–89, 2015 | 2004 |
| NALM-6 | PD | 19 | M | Pre-B-cell ALL | Peripheral blood | DSMZ | V^c^ | Int. J. Cancer 174-180, 1979 | 1984 |

D_X_ - Diagnosis

PD - Progressive Disease

ATCC - American Type Culture Collection

DSMZ- Deutsche Sammlung von Mikroorganismen und Zellkulturen

COGcell- Children's Oncology Group Cell Culture and Xenograft Repository ([www.COGcell.org](http://www.COGcell.org))

TXCCR- Texas Cancer Cell Repository ([www.TXCCR.org](http://www.TXCCR.org))

STR - Short Tandem Repeat

V - Cell line profile matched the STR from the original patient sample

V^C^ - Cell line STR matched to the STR profile available from a commercial STR database (ATCC and DSMZ)

* - year when the cell line was established or obtained

**Supplemental Table S2:** Primary clinical samples

| **Sample** | **Age (years)** | **Sex** | **Diagnosis** | **Source** | **Source** |
| --- | --- | --- | --- | --- | --- |
| Patient-1 | - | M | T-cell lymphoblastic leukemia | Peripheral blood | TXCCR |
| Patient-2 | 41 | M | T-cell lymphoblastic lymphoma | Peripheral blood | TXCCR |
| Patient-3 | 59 | M | T-cell lymphoblastic lymphoma | Bone marrow | TXCCR |
| Patient-4 | 40 | M | B-cell lymphoblastic lymphoma | Peripheral blood | TXCCR |

TXCCR – Texas Cancer Cell Repository

**Supplemental Table S3:** Primers for full length *CERS6*, *FAS* and *FAS* mutants

| **Construct** | **Primers** |
| --- | --- |
| CERS6^WT^ | F: 5’-AATTTAGCGATCGCCATGGCAGGAATCTTAGCCTGGTTCT-3'  R: 5’-AACCAATAACGCGTATCATCCATGGAGCAGGAGCCAGTCA-3’ |
| FAS^WT^ | F: 5’-AATAATTTAGCGATCGCCATGCTGGGCATCTGGACCCTCCTA-3’  R: 5’-CGCAACCACGCGTGACCAAGCTTTGGATTTCATTTCTG-3’ |
| FAS^Δaa174-236^ | F: 5’- pGCTGGAGTCATGACACTAAG-3’  R: 5’- GTTAGATCTGGATCCTTCC -3’ |
| FAS^ΔDD^ | F: 5’- pCTCAAGGACATCACTAGTGACTCAG-3’  R: 5’- TGCCACTGTTTCAGGATTTAAGGTTGGAG -3’ |
| FAS^Δaa315-335^ | F: 5’-AATAATTTAGCGATCGCCATGCTGGGCATCTGGACCCTCCTA-3’  R: 5’-CGCAACCACGCGTGATGATAGTCTGAATTTTCTC-3’ |
| FAS^C199V^ | F: 5’- pACAGTGAGAAAGCACAGAAAGG-3’  R: 5’- TTTCTGTACCTCCTTTCTCTTCACCC -3’ |
| FAS^Δaa174-314^ | F: 5’- pCTCAAGGACATCACTAGTGACTCAG-3’  R: 5’- GTTAGATCTGGATCCTTCC -3’ |
| FAS^Δaa174-236&315-335^ | F: 5’-AATAATTTAGCGATCGCCATGCTGGGCATCTGGACCCTCCTA-3’  R: 5’-CGCAACCACGCGTGATGATAGTCTGAATTTTCTC-3’  FAS^Δaa174-236^ was used as a template for PCR |
| FAS^aa174-314^ | F: 5’- AATAATGCAGCGATCGCCATGTTGGGGTGGCTTTGTCTTCTTC-3’  R: 5’-CGCAACCACGCGTGATGATAGTCTGAATTTTCTC-3’ |
