# Supplementary material for: Ceramide synthase-6 confers resistance to chemotherapy by binding to CD95/Fas in T-cell acute lymphoblastic leukemia
Source: Cell Death Dis. 2018 Sep 11;9(9):925. doi: 10.1038/s41419-018-0964-4 (PMC6133972; doi:10.1038/s41419-018-0964-4)
Supplement: Supplementary file 2 — Supplemental Figure Legends [file 41419_2018_964_MOESM2_ESM.docx]

**Supplemental Figure legends**

**Fig. S1** mRNA expression of CERS6 in various cancers relative to normal tissues.

*CERS6* expression levels in tumor samples were compared to normal tissues in different cancer types. *CERS6* expression levels were obtained from UCSC Xena (https://xenabrowser.net)

**Fig. S2** Increased apoptosis in ABT-737 treated ALL cells with *CERS6* knockdown.

Annexin-V apoptosis assay by flow cytometry (Related to Fig. 2d). Each of the quadrant represents a specific cell population. Quadrant I: Late apoptotic cells (Top right, Annexin-V positive and propidium iodide positive; blue), Quadrant II: Dead cells (Top left; green), Quadrant III: Live cells (Bottom left; Annexin-V negative and propidium iodide negative; pink) and Quadrant IV: Early apoptotic cells (Bottom right; Annexin-V positive and propidium iodide negative; orange). Apoptotic cells comprise of early apoptotic cells and late apoptotic cells added together.

**Fig. S3** No significant changes in BLC-2 family of proteins upon CERS6 knockdown or overexpression and *CERS6* knockdown cells are sensitive to dexamethasone (DXM), a standard drug used in ALL treatment

**a** Protein levels of BCL-2 family members in CCRF-CEM cells with CERS6 knockdown or overexpression **b** Dose response curves showing concentration of DXM on X-axis and survival fraction on Y-axis on a log_10_ scale. Bar graphs depict survival fraction at 100nM of DXM. **c** DXM showed higher levels of cleaved caspase-8 (left), cleaved PARP and cleaved caspase-3 (right) in CCRF-CEM cells with *CERS6* knockdown in comparison to cells transduced with NT-shRNA. GAPDH was used as a loading control.

**Fig. S4** FAS surface expression levels in cells with *CERS6* knockdown and overexpression

CCRF-CEM cells with *CERS6* knockdown or overexpression along with their respective controls were incubated with either FITC mouse anti-human IgG or CD95-FITC. FITC fluorescence intensity was increased in *CERS6* knockdown (2225 ±35.8 vs 206 ±52.3; p<0.001; n=3) in comparison to non-targeted shRNA while there was no significant change in FAS expression levels between cells with *CERS6* overexpression and cells transduced with the control vector (885 ±59.2 vs 737 ±7.2, n=3).
