# Supplementary figures and images for: Ceramide synthase-6 confers resistance to chemotherapy by binding to CD95/Fas in T-cell acute lymphoblastic leukemia
Source: Cell Death Dis. 2018 Sep 11;9(9):925. doi: 10.1038/s41419-018-0964-4 (PMC6133972; doi:10.1038/s41419-018-0964-4)

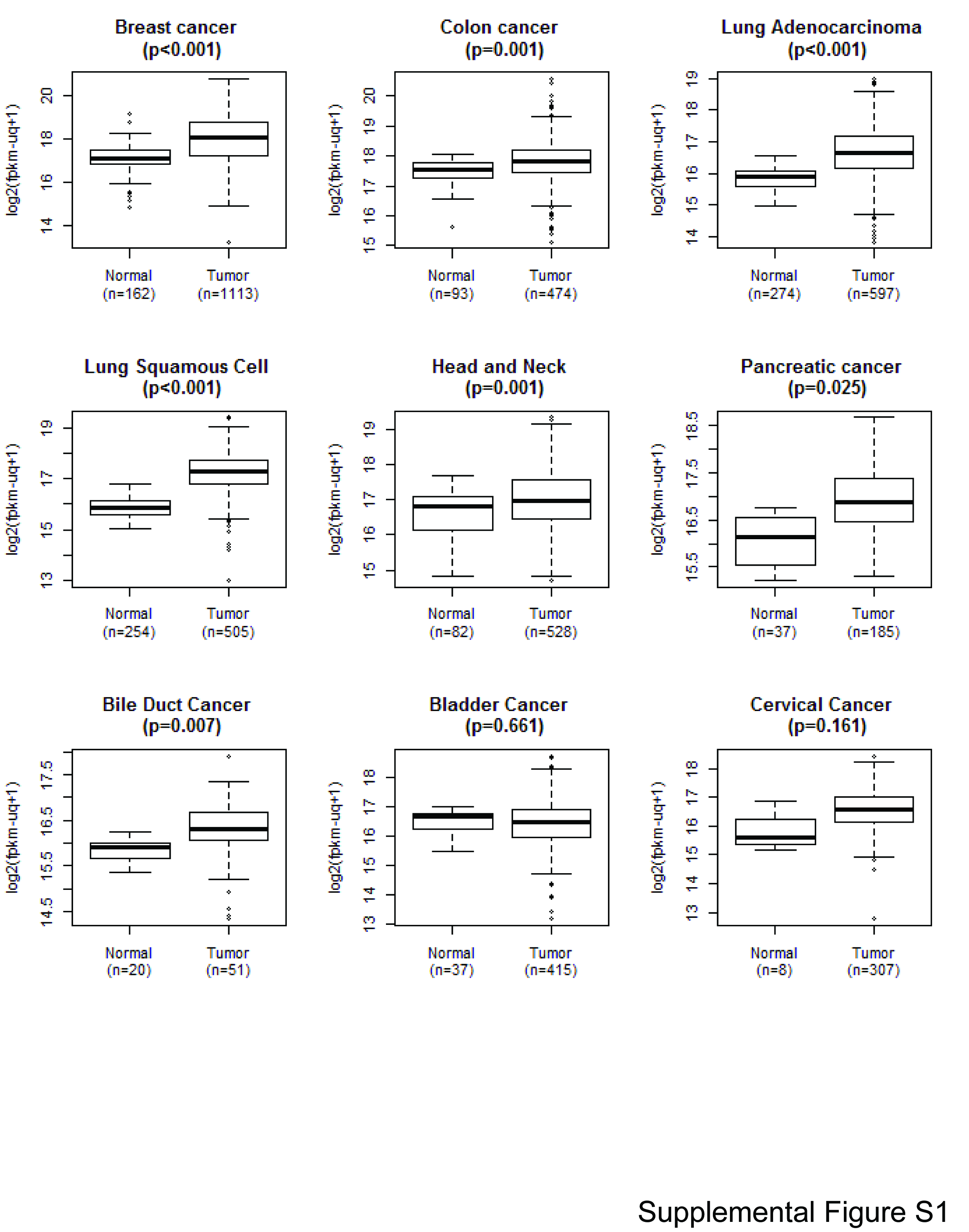

Supplement: Supplementary file 3 — Supplemental Figure 1 [file 41419_2018_964_MOESM3_ESM.tif]

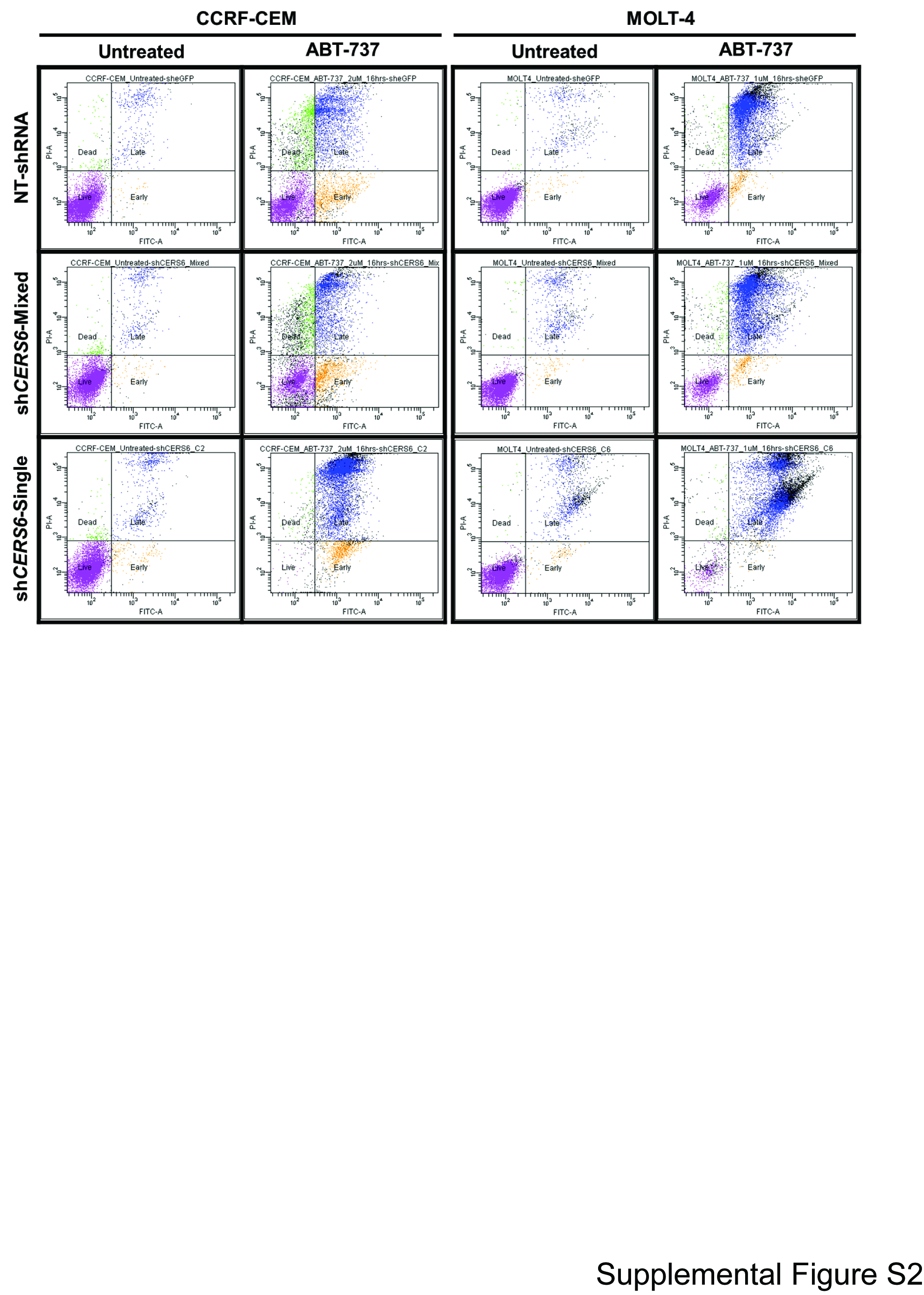

Supplement: Supplementary file 4 — Supplemental Figure 2 [file 41419_2018_964_MOESM4_ESM.tif]

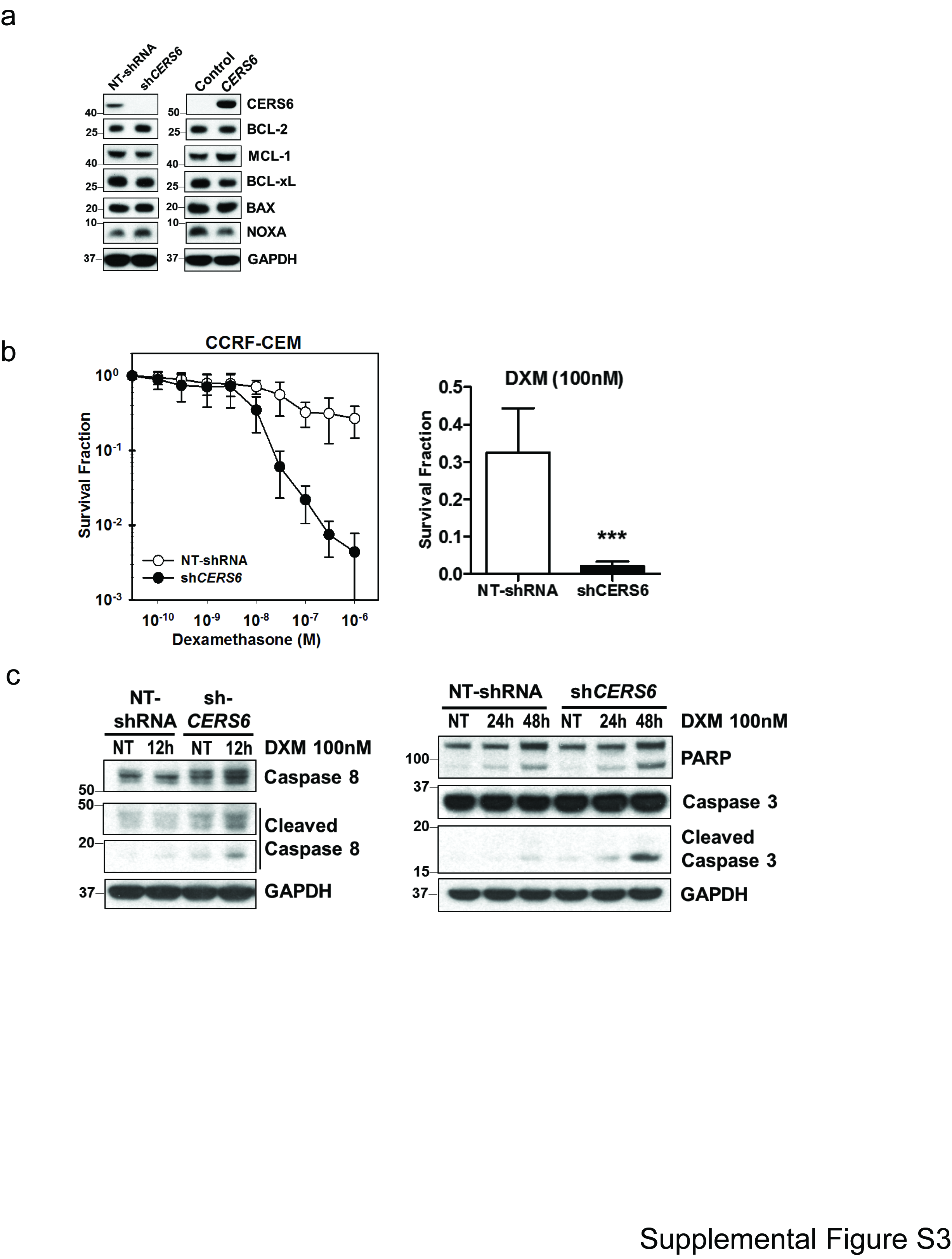

Supplement: Supplementary file 5 — Supplemental Figure 3 [file 41419_2018_964_MOESM5_ESM.tif]

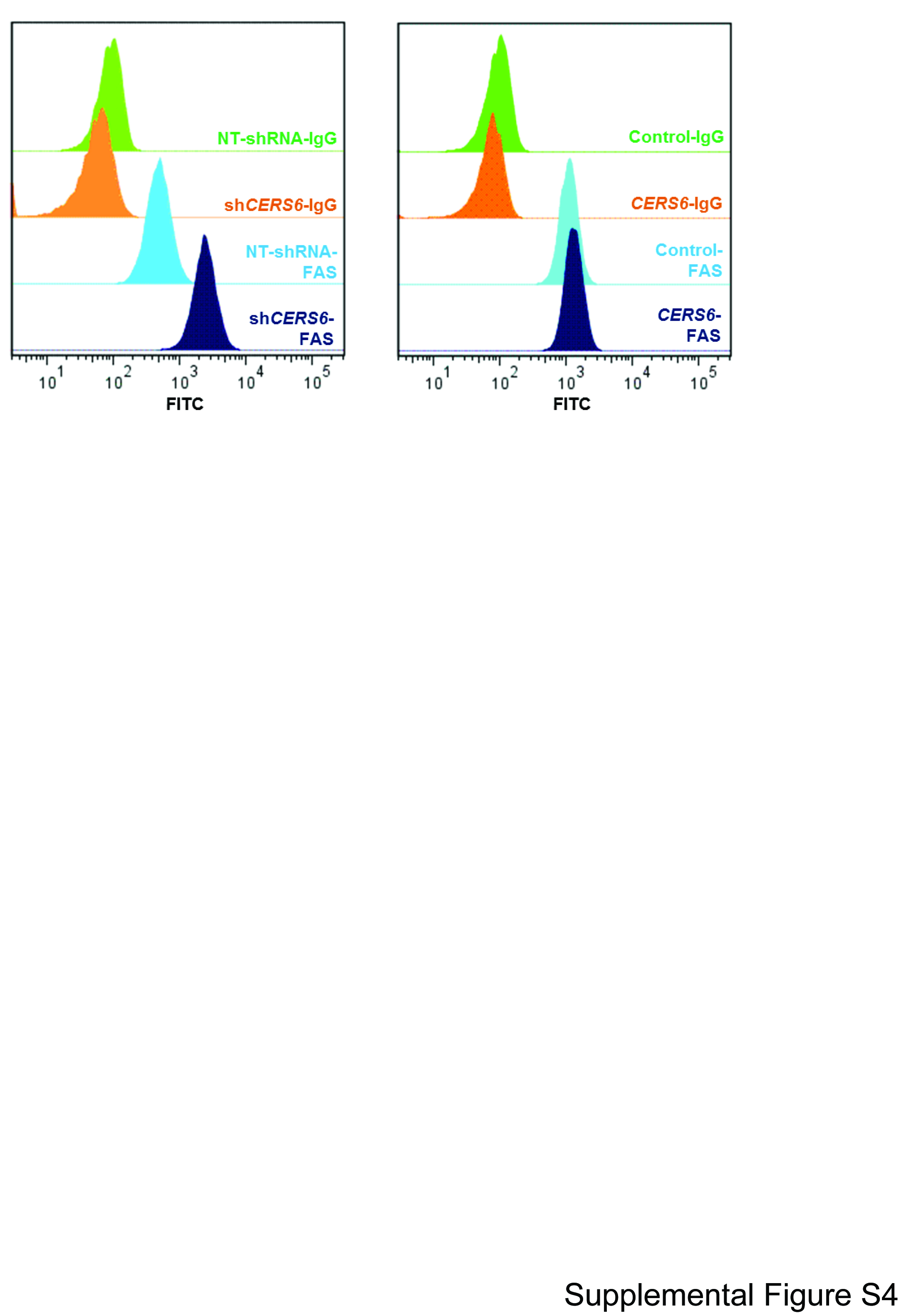

Supplement: Supplementary file 6 — Supplemental Figure 4 [file 41419_2018_964_MOESM6_ESM.tif]
